# Eye-Tracking Training Improves Inhibitory Control in Children with Attention-Deficit/Hyperactivity Disorder

**DOI:** 10.3390/brainsci11030314

**Published:** 2021-03-02

**Authors:** Tsz Lok Lee, Michael K. Yeung, Sophia L. Sze, Agnes S. Chan

**Affiliations:** 1 Department of Psychology, The Chinese University of Hong Kong, Shatin, New Territories, Hong Kong, China; tllee@link.cuhk.edu.hk (T.L.L.); lmsze@cuhk.edu.hk (S.L.S.); 2 Department of Rehabilitation Sciences, The Hong Kong Polytechnic University, Hong Kong, China; kin-chung-michael.yeung@polyu.edu.hk; 3 Research Center for Neuropsychological Well-Being, The Chinese University of Hong Kong, Shatin, New Territories, Hong Kong, China

**Keywords:** eye-tracking, inhibition, mental flexibility, cognitive training, ADHD

## Abstract

Disinhibition is a common sign among children with attention-deficit/hyperactivity disorder (ADHD). The present study examined the effect of computerized eye-tracking training to improve inhibitory control in ADHD children. Thirty-two ADHD children (mean age = 8.4 years) were recruited. Half of the participants underwent 240 min of eye-tracking training over two weeks (i.e., experimental group), while the other half did not receive any training (i.e., control group). After training, the experimental group exhibited significant improvements in neuropsychological tests of inhibition, such as faster reaction time in the incongruent condition of the Flanker test, more unique designs in the Category Fluency and Five-Point Tests, and a faster completion time in Trail 2 of the Children’s Color Trail Test. The control group did not show significant changes in any of these tests. Our findings support the use of eye-tracking training to improve the inhibitory control of ADHD children.

## 1. Introduction

Inhibitory control is the ability to resist distractor interference and cancel irrelevant responses [1], and poor inhibitory control is one of the common cognitive deficits observed in children with attention-deficit/hyperactivity disorder (ADHD). Children with response inhibition problems are likely to elicit conduct problems and act hyperactively [2], maintain a high child anxiety level [3], and achieve poorly in school [4]. Therefore, interventions that can effectively improve the inhibitory control of children with ADHD are clinically significant.

The eye-tracking technique has been developed since the 1950s, and involves the use of a device that can measure eye movement. This technique has a long history as a research tool and recently has been suggested for clinical application. The clinical application of eye-tracking to date has focused on the diagnosis of neurocognitive disorders, such as dementia of Alzheimer’s type [5,6] and ADHD [7,8]. Relatively little is known about the possibility of using an eye-tracking system for cognitive training [9].

The rationale of using an eye-tracking system for cognitive training is that eye movement abnormality has been commonly seen in patients with brain disorders, including Alzheimer’s disease [10,11,12], Parkinson’s disease [13,14], frontotemporal dementia [15,16], autism spectrum disorder [17,18], and ADHD [19,20]. In addition, abnormal eye movement in ADHD children has been proposed to be associated with frontal lobe impairment, such as disinhibition and perseveration [19,21]. Thus, eye-tracking-based cognitive training that aims to improve eye movement should be an effective intervention for improving frontal lobe function, such as inhibitory control. An eye-tracking-based computerized cognitive training program was developed by our team and has been applied clinically for a few years. The purpose of the present study was to examine the effect of this eye-tracking training program on inhibitory control in children with ADHD.

Computerized cognitive training for children with ADHD has been studied extensively [22,23,24,25]. However, most of the studies were on attention or working memory training, and relatively few studies were on enhancing inhibitory control, and the results have been not very encouraging. One study examined the effectiveness of computer-operated training on inhibition in a group of children. The results showed that the children’s performance after training was improved significantly only for the Progressive Matrices Test but not the go/no-go or Stroop tasks. These results indicated that after a three-week training program, children showed significant improvement in reasoning but not inhibitory control [26]. Similar results were reported in a study that employed computerized training for inhibition and working memory. Children with ADHD who underwent a 5-week training program showed improved attention but not inhibitory control ability [27]. In another study that utilized computerized training for a group of adults with ADHD (*n* = 60), the results showed that both the experimental and control groups showed improvements in working memory, inhibition, intrusions, response variability, and flexibility. The authors concluded that the computerized training they employed had no benefit for executive function compared to the control group [28].

Unlike conventional computerized training, this study’s training required participants to respond by their eyes instead of their hands. In addition, in contrast to the static pictures used in Johnstone et al.’s training [27], our training was animated so children would find the training more engaging. More importantly, our training aimed at improving the inhibitory control of ADHD children through gaze training, whereas Johnstone et al.’s training [27] focused on improving working memory and inhibition through motor inhibition tasks. Eye movement control is also associated with specific regions in the frontal lobes [29], such as the frontal eye fields [30], the supplementary eye fields [31], and the dorsolateral prefrontal cortex [32]. One previous study showed that the inhibitory control of ADHD children was modulated by the frontal lobes [33]. Therefore, our eye-tracking-based cognitive training was based on the premise that it strengthens the functioning of multiple sub-regions within the frontal lobes, resulting in improved inhibitory control. Although conventional computerized training has not yielded positive results, we hypothesized that eye-tracking-based computerized training might yield encouraging results.

## 2. Materials and Methods

### 2.1. Participants

Thirty-two children were recruited for this experiment. All of them received an ADHD diagnosis from a clinical psychologist or a psychiatrist in Hong Kong. The participants were divided half into the experimental and control groups, with matched demographics. There was an equal number of participants in both the experimental and control groups. The children were recruited through online advertisement, and the selection requirements were: (1) aged between 6 to 12 years, (2) attending mainstream schools, (3) normal or corrected-to-normal vision, and (4) being diagnosed with ADHD by a clinical psychologist or a psychiatrist, with all of them meeting the Diagnostic and Statistical Manual of Mental Disorders [34]. The sample size was determined based on Johnstone et al.’s study [27], in which a Cohen’s d of 1.28 was found for the improvement of ADHD symptom frequency in the high-intensity group. Using G*Power 3.1.9.7 [35], at least 11 participants per group are required to detect group differences to achieve a power of 0.8. Therefore, the present sample size (i.e., 16 participants per group) should be adequate. Children with any parent-reported history of stroke, head injury, mood disorder, and mental retardation were excluded from participating in the present study. All recruited participants did not participate in any treatments using the eye-tracking technique before and during the experiment.

### 2.2. Procedure

Before the training, the children’s inhibitory control was assessed. Simultaneously, the parents/guardians of the participants were asked to complete questionnaires about their children’s medical history and behavior. All participants in the experimental group completed the eye-tracking training in eight sessions over two weeks. Each session lasted about 40 min. During each eye-tracking training session, participants were given three 10-min eye-tracking training tasks, with a 5-min break in between training tasks. That is, the participants received 240 min of training in total. The eye-tracking-based training involved interaction primarily with the computer rather than the experimenter. Thus, the training could be easily applied in a group setting, and there was no substantial difference between individualized and group training in terms of the efficiency of training. In the present study, the training was conducted in groups of four to six participants, with eight groups per day; thus, group training was less labor-intensive and more cost-effective than individualized training. All of the participants received the training at the same location. For the control group, no training was given. The post-assessment was conducted, for both experimental and control groups, after two weeks. It followed the same testing procedure as the pre-assessment.

### 2.3. Computerized Eye-Tracking Training

The training was conducted with a Tobii Eye Tracker 4C (Tobii, Stockholm, Sweden), a 23” external monitor, and a desktop computer running Windows 7. The eye tracker was a non-contact tracker with a temporal resolution of 90 Hz and head tracking. Participants sat approximately 50 cm in front of the monitor, on which the eye tracker was placed. After a six-point calibration, participants engaged in the training. The computer training game was developed by the Pro-talent Association Ltd. (a non-profit organization) in Hong Kong. This program was developed based upon scientific evidence regarding eye gazing and frontal lobe processing to improve inhibitory control. It is well known that inhibitory control processes correlate with many other executive functions and attention processes. Therefore, although our eye-tracking-based cognitive training focused on inhibitory control, the cognitive processes involved were not restricted to inhibitory control processes but also included their related cognitive domains.

The training program consisted of six modules, and each module had three levels (i.e., easy, moderate, hard), which suited children with different inhibitory control abilities. The goal was the same across modules—to improve attention and impulse control—but the games’ themes varied. In each game, the participants were required to fixate on a target, and a score was given once they had fixated for long enough. For example, in one game, the participants had to fixate on a falling stone for long enough before it fell. In another module, the target was a rabbit. The fixation duration required to obtain the score increased as the level advanced. Each module’s training time was 10 min, and the participants could choose to continue or quit after each module. The program automatically proceeded to the next level when the child achieved a score of 80 or higher. Therefore, every participant needed to reach a particular performance criterion and complete the first level before moving on to the second level and so forth. Participants received positive feedback shown on the screen upon successful completion of each level. They did not know how many scores they got received throughout the training. Therefore, they were not able to socially compare their performance with others.

### 2.4. Tests and Materials

#### 2.4.1. Conners’ Parent Rating Scale-Revised: Short Form (CPRS-R:S)

The Chinese version of the CPRS-R:S [36] was used to assess ADHD symptoms in terms of the frequency of typical ADHD behaviors. Participants’ parents or guardians were asked to rate the frequency of their child’s typical ADHD behaviors (e.g., inattention, fidgeting) on a scale from 0 (not true at all) to 3 (very much true). The scale contains 27 items, and part of the items were summed into the oppositional, inattention, and hyperactivity scores. Each score ranged from 0 to 18. The higher the score, the higher the severity of the child’s ADHD symptoms. The scale has good internal consistency with Cronbach’s alpha >0.85 [36].

#### 2.4.2. Eriksen Flanker Test

We employed the Eriksen Flanker Test [37] to assess inhibitory control, a common test paradigm for evaluating children’s inhibitory abilities [38,39,40]. In each trial, a horizontal array of arrows was first shown at the center of a computer screen, and participants were instructed to judge the direction of the central arrow and to respond as quickly as possible by pressing the left (right) button of the mouse for the left (right) direction. The direction of the central target was equiprobable. The Flanker Test consisted of congruent and incongruent conditions. In the congruent (incongruent) condition, the flanker and target stimuli pointed in the same (opposite) direction. The flanker and target stimuli were then followed by a randomly selected inter-stimulus interval between 1700 and 2400 ms for each trial. A total of 100 trials were given. The whole test took around 5 min for everyone. Before the actual experiment, each participant underwent a practice session to become familiar with the test. The accuracy and mean reaction time in both conditions were calculated for analysis.

#### 2.4.3. Neuropsychological Tests for Inhibitory Control

Three paper-and-pencil tests were administered to assess inhibitory control. These tasks also targeted and assessed working memory and sustained attention in addition to the ability to resist perseveration (i.e., inhibition): (1) The Cantonese version of Category Fluency Test [41]. The participants were instructed to generate as many words as possible that belong to a specified category within a time limit of 1 min per category. (2) Five-Point Test [42]. A sheet of paper with a 5 × 8 five-dot matrix was given to the participants. Participants were asked to create as many unique patterns as possible by connecting two to five dots in each matrix using straight lines and without repetitions. The number of unique items drawn was scored. (3) Children’s Color Trails Test (CCTT [43]). This test consisted of two parts. In part one, participants were instructed to connect 15 encircled numbers in ascending order. In part two, they were asked to join the numbers in ascending order while alternating between colors. Time to completion was the dependent variable.

### 2.5. Data Analysis

The demographic features, ADHD symptoms, and baseline task performances between the experimental and control groups were compared using independent-sample *t*-tests and chi-squared tests. Then, we performed ANCOVA, repeated-measures ANOVA, and paired *t*-tests to evaluate task performance changes between post-assessment and baseline for each group. All statistical tests were two-tailed and performed using SPSS 24.0 software (IBM Corporation, Armonk, NY, USA).

## 3. Results

The demographics, ADHD symptoms, and baseline assessment results of the experimental and control groups are shown in Table 1. No significant differences between the two groups were found in any variables at baseline (*p*s from 0.15 to 1.00).

### 3.1. Improvement in the Flanker Test after Training

**Figure 1:**
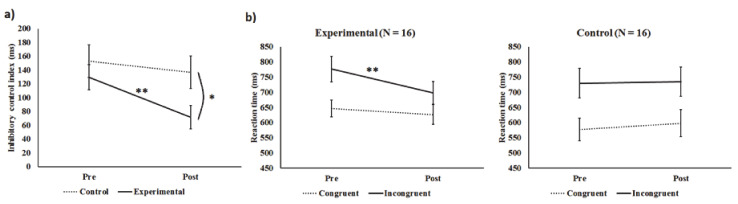
(**a**) Inhibitory control index. (**b**) Mean reaction time in the experimental (*n* = 16) and control groups (*n* = 16). “Pre” and “Post” refer to the test performance of participants before and after real training/no training period, respectively. Error bars represent 1 standard error ± the mean. Asterisks indicate the level of significance of t-tests (two-tailed). * *p* < 0.05, ** *p* < 0.01.

To illustrate the effect of eye-tracking training on inhibition, an inhibitory control index was calculated by subtracting mean reaction time in the congruent condition from that in the incongruent condition, with a lower index indicating a better inhibitory control ability (Figure 1a). An ANCOVA with the factor group (experimental vs. control), post-test scores as the dependent variable, and pre-test scores as the covariate was performed, and the result showed a significant group difference in the inhibitory control index at post-assessment after covarying its pre-assessment score (*F*(1, 29) = 6.4, *p* = 0.017, $\eta_{p}^{2}$ = 0.18). This finding suggested the effectiveness of computerized eye-tracking training on inhibitory control. Next, to clarify the basis of the change in the inhibitory control index, the changes in the mean congruent and incongruent flanker reaction time of the experimental and control groups were examined (Figure 1b). A three-way mixed ANOVA was performed with the condition (congruent, incongruent) and time (pre, post) as within-subjects factors, and group (experimental, control) as a between-subjects factor. The group × time interaction was significant (*F*(1, 30) = 4.33, *p* = 0.046, $\eta_{p}^{2}$ = 0.13), although the group × condition × time interaction was not, *p* = 0.17. Therefore, separated ANOVAs were conducted in both groups to evaluate the treatment effect. As expected, there was a significant condition (congruent, incongruent) × time (pre, post) interaction in the experimental group, (*F(*1, 15) = 9.98, *p* = 0.006, $\eta_{p}^{2}$ = 0.40), but not in the control group (*F*(1, 15) = 0.48, *p* = 0.50, $\eta_{p}^{2}$ = 0.03). Paired t-tests showed that only the experimental group, (*t*(15) = 3.70, *p* = 0.002, *d* = 0.93), but not the control group (*t*(15) = 0.18, *p* = 0.86, *d* = 0.04), exhibited a significant decrease in mean reaction time in the incongruent condition. No significant change was found for mean reaction time in the congruent condition in either the experimental (*t*(15) = 0.77, *p* = 0.45, *d* = 0.19) or the control group (*t*(15) = 1.34, *p* = 0.20, *d* = 0.33).

Changes in the flanker accuracy were also examined in a similar way using repeated-measures ANOVA. The condition × time interaction was not significant in either the experimental group (*F*(1, 15) = 3.19, *p* = 0.09, $\eta_{p}^{2}$ = 0.33), or the control group (*F*(1, 15) = 1.63, *p* = 0.22, $\eta_{p}^{2}$ = 0.098). Paired t-tests also showed that neither the experimental group (*t*(15) = 1.34, *p* = 0.20, d = 0.33) nor the control group (*t*(15) = 0.82, *p* = 0.42, *d* = 0.21) exhibited significant improvement in accuracy in the incongruent condition. No significant change was found for accuracy in the congruent condition in either the experimental (*t*(15) = 0.76, *p* = 0.46, *d* = 0.19) or the control group (*t*(15) = 0.71, *p* = 0.49, *d* = 0.18).

Overall, these findings show that only ADHD children who received the eye-tracking training exhibited specific improvement in inhibitory control, indicated by a significant improvement in the inhibitory control index.

### 3.2. Improvement in the Neuropsychological Tests after Training

**Figure 2:**
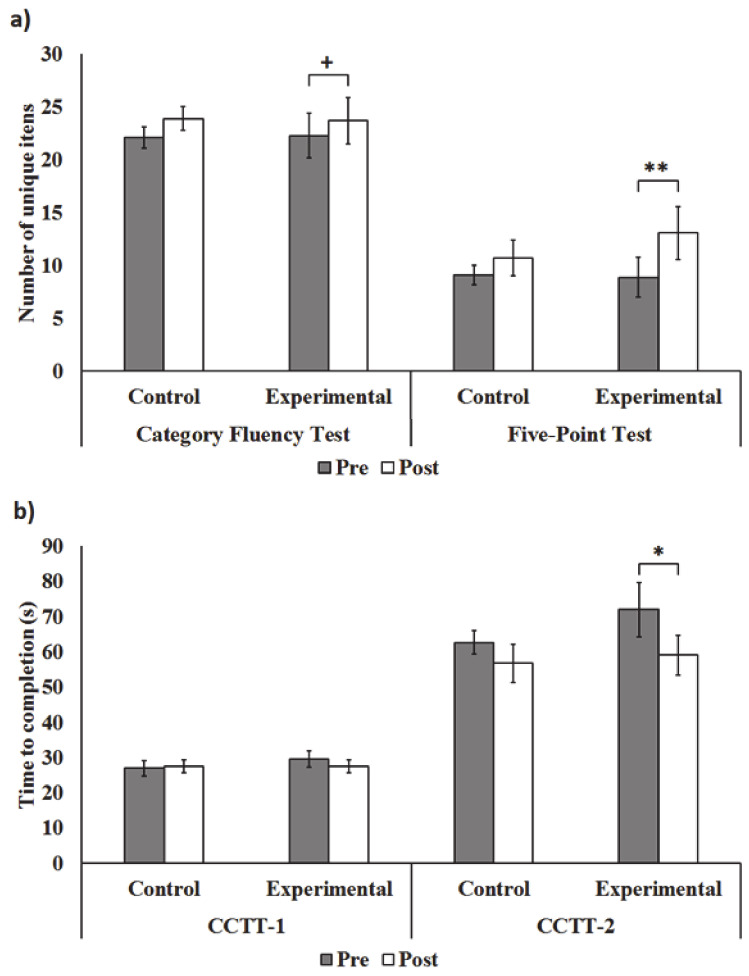
Performance in the (**a**) Category Fluency Test and Five-Point Test, in addition to the (**b**) Children’s Color Trails Test (CCTT) in the experimental (*n* = 16) and control (*n* = 16) groups. “Pre” and “Post” refer to the test performance of participants before and after real training/no training period, respectively. Error bars represent 1 standard error ± the mean. Asterisks indicate the level of significance of paired *t*-tests (two-tailed). ** *p* < 0.01, * *p* < 0.05, ^+^ *p* = 0.05.

Next, changes in the neuropsychological test performance for inhibitory control were examined for the experimental and control groups using paired t-tests (Figure 2). The ANCOVA did not show a significant group difference in post-assessment performance after covarying its pre-assessment scores (*p*s > 0.16). For the Category Fluency Test, only the experimental group (*t*(15) = 2.09, *p* = 0.054, *d* = 0.52), but not the control group (*t*(15) = 1.29, *p* = 0.22, *d* = 0.32), produced significantly more unique words at post-assessment compared to baseline. Despite the seemingly larger standard deviations in the pre- and post-assessment in the experimental group, there was a considerably larger standard error of the difference between means, which lowered the *t*-statistics, in the control group (i.e., 1.46) compared to the experimental group (i.e., 0.70). Thus, the significant change in the experimental group was due to more consistent improvement in task performance (i.e., individuals improving to a more similar extent). Similarly, for the Five-Point Test, the experimental group created significantly more unique designs (*t*(15) = 3.23, *p* = 0.006, *d* = 0.81), whereas the control group did not (*t*(15) = 1.21, *p* = 0.25, *d* = 0.30). In addition, we found that only the experimental group (*t*(15) = 2.15, *p* = 0.048, *d* = 0.54), but not the control group (*t*(15) = 1.26, *p* = 0.23, *d* = 0.32), was significantly faster to complete the CCTT-2. Neither group exhibited significant changes in the time to complete the CCTT-1 (experimental: *t*(15) = 0.81, *p* = 0.43, *d* = 0.20; control: *t*(15) = 0.33, *p* = 0.74, *d* = 0.08). Consistent with the Flanker Test results, these results indicate that only ADHD children who underwent the eye-tracking training exhibited specific improvement in inhibitory control.

## 4. Discussion

This study examined the effectiveness of computerized eye-tracking training on inhibitory control in ADHD children. We found that only children who received 240 min of training over two weeks scored a significantly lower inhibitory control index in the Flanker Test, generated significantly more unique items during the Category Fluency Test and the Five-Point Test, and were significantly faster to complete the CCTT-2. More importantly, for the flanker task, we found that the group difference in the inhibitory control index remained significant after controlling for baseline performance. These results suggest that the treatment effect was not due to the baseline difference in this index.

The positive treatment effects are consistent with those reported by a previous study investigating the therapeutic benefits of eye-tracking training on visual attention [44]. With three weeks of training, ADHD children aged 8 to 15 years showed improvement in visual attention when trained using an eye-tracker, but no improvement was found to those trained using a computer mouse. Our findings are thus in keeping with these previous findings that eye-tracking training may benefit ADHD children. More importantly, this study demonstrated that the treatment effect was extended to inhibitory control and that such an effect could be found in children at a younger age. These suggest a possibility of using eye-tracking-based computerized training for early intervention. In addition, it should be noted that the effect in the present study was observed after a relatively short duration of training; that is, the training was administered for only eight sessions at 40 min per session. In our clinical observation, progressive improvement has been observed over months or even years. Therefore, further studies on the long-term effect of this training should be interesting.

Although finger-control computerized training has failed to elicit significant improvement on inhibitory control in children with ADHD [27,28,45], the present study demonstrated a significant improvement in various executive functions after two weeks of training. In the traditional form of training, a mouse or a finger is used, whereas in eye-tracking training, the eyes are used as the medium with which to play games. The difference in the element used may contribute to the different training effects. Given that an eye gaze deficit has been observed in children with inhibitory control problems, such as those with ADHD [33,46,47], the participants were trained in controlling their eye movements instead of hand movements to complete the tasks. Further investigation should be done on the eye gaze improvement after training, and the relationship between the behavioral and neurophysiological changes should also be evaluated. For instance, regions in the frontal lobe, such as the frontal eye fields, supplementary eye fields, and the dorsolateral prefrontal cortex, are responsible for the initiation [30], regulation [31], and executive control [32] of saccade. More investigation is needed to evaluate the functional improvements in these regions.

If the treatment effect is also due to interaction with other participants and frequent exposure of the training environment, then one would expect a general improvement in task performance. Instead, we found that the experimental group demonstrated a specific improvement in inhibitory control as evidenced by improved reaction time in only the incongruent, but not the congruent, condition of the flanker task, thus rendering these explanations unlikely. Because our cognitive training was designed to improve inhibitory control, such a condition-specific improvement was likely the consequence of the training. In addition, in terms of the response mode required and the stimuli used, the training tasks were considerably different from the assessment tasks used in this study. Notably, while the training tasks were performed using eye gaze, the assessment tasks were completed by means of the finger or upper limb movement or oral production. Therefore, the treatment effect observed in the experimental group was unlikely due to mere practice effects.

Because this eye-tracking training is cost-effective, with a short training time (i.e., around 240 min) and a relatively inexpensive eye-tracking device, it may serve as a cost-effective intervention to be implemented in class or at home for ADHD children. However, the transfer effects of this training on daily life remain uncertain. The current study results showed improvements on several neuropsychological tests, but changes in daily functioning and school performance are still unknown. In addition, the optimal number of training hours needed is unclear. In this study, 240 min of training was given, and improvements were found. Future investigations should focus on determining the training time needed to maximize the effect of this intervention in order to develop an efficient training protocol. In addition, the present study only examined the immediate effect of eye-tracking training. Follow-up studies should be conducted to determine whether the training can produce a long-lasting effect.

A recent meta-analysis synthesized data from cognitive intervention studies that employed passive and active control groups [48]. This article performed two meta-analyses to compare the effect size differences between studies that used passive control groups and active control groups. The first analysis summarized the effect sizes of cognitive training meta-analyses. Across meta-analyses, cognitive training studies with passive controls yielded an effect size, *d* = 0.030, that is larger than studies with active controls. In the second analysis, which was a double-controlled meta-analysis of synthesized studies that simultaneously employed both active and passive controls, the results showed that the performance differences between active and passive controls were negligible and not significant (*g* = 0.058). The above results suggest that an active placebo control group did not have a meaningful performance difference compared to the passive group. Therefore, the significant results in the present study were unlikely due to the non-specific effects induced by the training program. However, further studies with an active control group may be useful to specify which component of the training yielded the significant treatment effects.

The computerized training utilized eye-tracking equipment as a way for intervention in the present study. Specifically, participants were trained to use their gaze to accomplish different goals, in which they must be able to voluntarily resist distractor interference and inhibit unwanted responses. Unfortunately, the eye-tracker used in this study was for gaming purposes, hence it could not store any eye-tracking data that were extractable for research use. Therefore, the lack of eye-tracking data that would characterize changes in eye movement throughout the two-week training poses a limitation to the present study. Despite this, our recent study using a different ADHD sample while employing an active control group found that only the participants who underwent the eye-tracking training showed significant improvement in the saccade latency and accuracy in the anti- and pro-saccade tasks, respectively [49].

The Eriksen Flanker Test primarily assessed cognitive inhibition as participants resisted distraction induced by competing or distracting stimuli. By comparison, the Category Fluency Test, Five-Point Test, and the Children’s Color Trails Test similarly drew on cognitive and motor inhibition because both tests required participants to suppress the oral/motor response they had made while at the same time resisting perseveration on the same cognitive set or task. Because eye-tracking data were not available, we could not examine the relationship between specific eye movement indicators and particular aspects of inhibitory control. Because inhibitory control is a multifaceted construct [50], future research would benefit from mapping changes in eye movement indicators (during the two-week training and the assessment) after eye-tracking training in ADHD. In addition, further studies would benefit from investigating the treatment effects on children of different ages, genders, and subtypes of ADHD.

## 5. Conclusions

This study demonstrated that 240 min of eye-tracking training significantly improved the inhibitory control of children with ADHD. These findings highlight the importance of the eye-tracking component in cognitive training. Computerized eye-tracking training may serve as a potential intervention to improve frontal lobe functioning in children with special educational needs or attentional problems.

## Figures and Tables

**Table 1:** Demographics, clinical symptoms, and baseline task performance of the experimental (*n* = 16) and control groups (*n* = 16).

	Control (*n* = 16)		Experimental (*n* = 16)			
Variables	*M*	*SD*	*M*	*SD*	*t/χ2*	*p*
Age (yr)	8.5	1.4	8.3	1.6	0.47	0.65
Gender (F/M) ^#^	3/13		3/13		0.00	1.00
Handedness (L/R) ^#^	2/14		0/16		2.13	0.14
**CPRS-R:S ^@^**						
Oppositional	12.5	4.3	10.7	3.9	1.21	0.24
Cognitive problems/ Inattention	13.9	3.7	13.3	3.9	0.45	0.66
Hyperactivity	10.3	2.4	9.4	5.5	0.63	0.53
ADHD index	27.7	4.7	26.6	6.1	0.56	0.58
**Flanker Test**						
**Congruent condition**						
Accuracy (%)	88.4	6.7	83.9	12.0	1.31	0.20
Reaction time (ms)	576.9	149.6	646.8	112.1	1.50	0.15
**Incongruent condition**						
Accuracy (%)	58.8	19.5	60.4	20.9	0.23	0.82
Reaction time (ms)	729.8	195.9	776.3	168.5	0.60	0.55
**Category Fluency Test**						
Number of unique words	22.1	4.2	22.3	8.4	0.11	0.92
**Five-Point Test**						
Number of unique designs	9.1	3.6	8.9	7.5	0.09	0.93
**Children’s Color Trails Test**						
**Trail 1**						
Completion time (s)	27.0	8.8	29.6	9.2	0.81	0.42
**Trail 2**						
Completion time (s)	62.7	13.4	72.0	30.8	1.11	0.28

*Note.* CPRS-R:S = Conners’ Rating Scale for Parents-Revised: Short Form. ^#^ Chi-squared tests were used to compare groups. ^@^ One missing data in the control group.

## Data Availability

The data and code that support the findings of this study are available from the corresponding author upon reasonable request.
